# Association

**Published:** 1868-07-15

**Authors:** 


### EDITORIAL.

### *Association.*

In order to advance the real value of the JOURNAL to its readers, the editor has secured as permanent associate, WALTER HAY, M.D., whose valuable translations have from time to time appeared in the JOURNAL. He will assume the task of translations of valuable papers from foreign medical journals, and in each No. furnish an epitome of all that attracts professional attention in the Old World. It is unnecessary to say that Dr. Hay is one of the most accomplished medical scholars in this city, and the editor felicitates himself on having secured his association.

It is known to our readers as the intention of the JOURNAL to furnish exclusively *original*, or otherwise unattainable matter. Our associate will assist in securing this object. Any thing of professional interest which transpires in Europe will be furnished in our pages earlier than by any other medium on the Continent. The CHICAGO MEDICAL JOURNAL proposes to make any sacrifice to secure the *latest* and *best* for its readers.

